# Integrating Genomic Selection and a Genome-Wide Association Study to Improve Days Open in Thai Dairy Holstein Cattle: A Comprehensive Genetic Analysis

**DOI:** 10.3390/ani15010043

**Published:** 2024-12-27

**Authors:** Akhmad Fathoni, Wuttigrai Boonkum, Vibuntita Chankitisakul, Sayan Buaban, Monchai Duangjinda

**Affiliations:** 1 Department of Animal Science, Faculty of Agriculture, Khon Kaen University, Khon Kaen 40002, Thailand; akhmad.f@kkumail.com (A.F.); wuttbo@kku.ac.th (W.B.); vibuch@kku.ac.th (V.C.); 2 Department of Animal Breeding and Reproduction, Faculty of Animal Science, Universitas Gadjah Mada, Yogyakarta 55281, Indonesia; 3 Network Center for Animal Breeding and Omics Research, Faculty of Agriculture, Khon Kaen University, Khon Kaen 40002, Thailand; 4 Department of Livestock Development, Bureau of Animal Husbandry and Genetic Improvement, Pathum Thani 12000, Thailand; buaban_ai@hotmail.com

**Keywords:** genomic selection, days open, Thai–Holstein crossbreeds, ssGAIREML

## Abstract

Days open (DO) is a critical economic and reproductive trait that is commonly employed in genetic selection. Making improvements using conventional genetic techniques is exceedingly challenging. Therefore, new techniques are required to improve the accuracy of genetic selection using genomic data. This study examined the genetic approaches of traditional AIREML and single-step genomic AIREML (ssGAIREML) to assess genetic parameters and the accuracy of estimated breeding values while also investigating SNP regions associated with DO and identifying candidate genes through a genome-wide association study (GWAS). The dataset included 59415 DO records from 36368 Thai–Holstein crossbred cows and 882 genotyped animals. The cows were classified according to their Holstein genetic proportion (breed group, BG) as follows: BG1 (>93.7% Holstein genetics), BG2 (87.5% to 93.6% Holstein genetics), and BG3 (<87.5% Holstein genetics). AIREML was utilized to estimate genetic parameters and variance components. The results of this study reveal that the average DO values for BG1, BG2, and BG3 were 97.64, 97.25, and 96.23 days, respectively. The heritability values were estimated to be 0.02 and 0.03 for the traditional AIREML and ssGAIREML approaches, respectively. Depending on the dataset, the ssGAIREML method produced more accurate estimated breeding values than the traditional AIREML method, ranging from 40.5 to 45.6%. The highest values were found in the top 20% of the dam dataset. For the GWAS, we found 12 potential candidate genes (*DYRK1A*, *CALCR*, *MIR489*, *MIR653*, *SLC36A1*, *GNA14*, *GNAQ*, *TRNAC-GCA*, *XYLB*, *ACVR2B*, *SLC22A14*, and *EXOC2*) that are believed to have a significant influence on days open. In summary, the ssGAIREML method has the potential to enhance the accuracy and heritability of reproductive values compared to those obtained using conventional AIREML. Consequently, it is a viable alternative for transitioning from conventional methodologies to the ssGAIREML method in the breeding program for dairy cattle in Thailand. Moreover, the 12 identified potential candidate genes can be utilized in future studies to select markers for days open in regard to dairy cattle.

## Simple Summary

Generally, improving the reproductive traits of livestock is challenging. Therefore, the United States and Europe have employed several new methods to improve these traits in dairy cattle. Genomic selection and genome-wide association studies (GWASs) are the most recent and reliable methods for enhancing dairy cattle reproduction. However, the application of these methods to crossbred dairy cattle in Asia has provided limited insights and results. To establish their effectiveness, comprehensive testing is essential. Therefore, these methods should be rigorously evaluated to confirm their suitability and benefits for tropical dairy cattle.

## 1. Introduction

Thailand has been attempting to create a tropical dairy breed using *Bos taurus* breeds (such as Holstein, Jersey, Brown Swiss, and Red Dane) and *Bos indicus* breeds (such as Sahiwal, Red Sindhi, Brahman, and Thai Native) for nearly 60 years. Holstein Friesian (HF) was chosen as the most common breed based on its milk production and suitability for Thailand’s socioeconomic conditions. More than 90% of dairy cattle in Thailand are a result of the crossing of HF with Sahiwal or Thai Native breeds. The crossing of these two breeds was intended to increase milk production from *Taurine* while incorporating resilience to heat, ticks, and tropical diseases. In addition, upgrading through the use of Holstein genetics has been implemented for decades to enhance milk production. The majority of the population in Thailand nowadays consists of dairy crossbreeds with ≥87.5% HF genetics [1]. 

Low reproductive performance is a significant factor in the culling of dairy cattle from a herd as it directly affects the profitability of dairy farmers. Both genetic and environmental factors cause variations in cattle reproduction. Moreover, global warming and heat stress are increasingly significant concerns in regard to cattle production because of their potentially severe impact on reproductive traits [2]. The genetic enhancement of reproductive traits is currently attracting significant attention regarding the genetic assessment of dairy cattle due to the impact of these qualities on milk production. The challenges in quantifying these characteristics and limited genetic diversity contribute to a slow response to selective breeding [3,4]. 

Days open (DO), a reproductive trait of economic significance, is widely used in genetic selection because of its recording simplicity [5,6]. The DO for Thai Holstein crossbred dairy cattle have previously been reported to be 124–187 days [5]. This value is greater than the DO value for Czech Holstein cows, Iranian Holstein cows, and Spanish Dairy Cattle [7,8,9] and is equal to the DO value for Ethiopian Holstein [10]. The area and the calving month can influence the variability of DO among dairy cattle [11]. Most reproductive traits have low heritabilities [12,13,14]. DO heritability for Thai dairy cattle has been previously reported to be in the range of 0.05–0.07 [5,15]. The same value was found for Ethiopian Holstein, Czech Holstein, Iranian Holstein, and Spanish Dairy cows [7,8,9,10]. Non-genetic factors are the primary cause of low heritabilities in reproductive traits, particularly farm management techniques that significantly affect reproductive performance [4]. Consequently, breeders face difficulties identifying techniques for enhancing reproductive traits in breeding programs.

A promising approach to enhancing the accuracy of selection involves implementing the genomic selection method introduced by Meuwissen et al. [16] and Hayes et al. [17]. In this method, the phenotypic, pedigree, and DNA information of single-nucleotide polymorphisms (SNP) is used to assess genomic estimated breeding values (GEBVs) [17]. This single-step genomic technique is a prevalent and extensively utilized method for genomic selection that integrates data from pedigree relationships and genetic information into a unified analysis. This technology decreases the time required to choose a superior animal and allows the selection of young animals based on DNA information. The enhancement in the reliability of GEBVs compared to the parental average EBV for reproductive traits ranged from 7 to 9% [18]. By selecting animals with exceptional genetic merit at a younger age with reasonable accuracy, the expenses of raising bulls at test stations can be decreased, resulting in a higher rate of genetic gain than that allowed by the old genetic evaluation approach.

Furthermore, the availability of SNPs makes it possible to implement genome-wide association studies (GWASs) as an alternative approach to identifying genomic regions associated with low heritability traits, such as reproductive traits [19]. The GWAS was introduced by Risch and Merikangas in 1996 as a method for investigating the genetic foundation of complex human disorders [20]. The fundamental principle of a GWAS is the occurrence of a linkage disequilibrium (LD) resulting from recombination during the extended duration of development of wild populations [21]. The GWAS have been extensively employed to identify quantitative trait loci (QTL) in cattle, including for complex traits [22]. Consequently, numerous potential genes have been identified to determine the reproductive characteristics of cattle [19]. Other studies applying the GWAS method to the reproductive characteristics of cattle have been conducted [23,24].

However, there are only a few previous genomic studies on the reproductive traits of dairy cattle [25,26,27,28]. Regarding Thai–Holstein crossbreeds, studies on genomic selection for reproductive traits have only been conducted for the number of services per conception (NSPC) [29]. However, GWAS investigations on Thai dairy cattle have exclusively focused on milk yields, somatic cell score, and fat yields [30]. Hence, this study aimed to estimate genetic parameters for DO and assess the accuracy of predicted breeding values using two different methods: the traditional AIREML method and the ssGAIREML approach for genomic selection. A GWAS was also used to identify the SNP loci influencing DO and investigate their effect on specific traits. It is expected that the findings of this study will be taken into account in order to modify the approach used in breeding projects for crossbred dairy cattle in tropical areas such as Thailand.

## 2. Materials and Methods

### 2.1. Phenotypic, Pedigree, and Genomic Data

A dataset consisting of 59,415 days-open (DO) records for 36,368 Thai–Holstein crossbred cows collected between 1996 and 2017 was supplied by the Bureau of Biotechnology in Livestock Production of the Department of Livestock Development, Thailand. DO data were described as the interval between calving dates and successful conception. Cows with unclear information for each record’s date, identity data, and more than one date of birth per parity or an implausible time gap between calving date and conception were excluded.

The cows were categorized based on the proportion of Holstein genetics (breed group, BG) as follows: BG1, cows exceeding 93.7% Holstein genetics; BG2, cows with 87.5% to 93.6% Holstein genetics; and BG3, cows possessing less than 87.5% Holstein genetics. The age at calving was classified into seven groups. Specific statistical information regarding the analyzed characteristics is presented in Table 1.

The pedigree dataset comprised 79,071 animals born between 1994 and 2016. Additionally, genotyping was conducted on 882 animals using the Illumina BovineSNP50 Bead Chip produced by Illumina Inc. in San Diego, CA, USA. Quality control was applied to the animals and markers consistent with the subsequent criteria: the minimum call rate for each marker was 90%, and the minor allele frequency was greater than 5%. Markers and animals that did not match these standards for quality control were removed. PREGSF90 software was utilized to analyze SNPs and assess sample quality control (QC) [31]. Following QC, 46,852 high-quality single-nucleotide polymorphisms (SNPs) from 868 samples were selected for GWAS analysis. 

### 2.2. Statistical and Genetic Analysis

SAS Studio online v.3.81 was used to validate and analyze days-open (DO) data for least squares means. Significant differences were compared by breed group and parity using Scheffe’s (*p* < 0.05) post hoc test in the generalized linear model for an unbalanced analysis of variance (GLM procedure). The variance components, heritability, and breeding value were estimated using the repeatability animal model in genetic analysis. The DO values that occurred in various parities were regarded as recurrent observations. Consequently, the model included the constant values of fixed effects, the effects of herd x year of calving, animals’ additive genetic effects, permanent environmental impacts (p), and residual effects (e), as follows:

***y*** = ***Xb*** + ***Qh*** + ***Zu*** + ***Wp*** + ***e***

where ***y*** is the trait of interest (DO); *b* denotes the fixed effects of breed group (3 levels), parity (5 levels), and year-month of calving (197 levels); ***h*** denotes the random effects of herd-year of calving (11,675 levels); ***u*** denotes the random additive genetic effects vector; ***p*** denotes the random permanent environmental effects vector; *e* denotes the random residual effects vector; and ***X***, ***Q***, ***Z***, and ***W*** represent the incidence matrix of fixed effects, random effects, additive genetic, and permanent environmental effects, respectively. The assumed covariance structures are as follows.

$$\mathrm{Var}\;\left[\begin{array}{c} h\\ u\\ p\\ e \end{array}\right]=\left[\begin{array}{cccc} I\sigma_{h}^{2} & 0 & 0 & 0\\ 0 & A\sigma_{u}^{2} & 0 & 0\\ 0 & 0 & I\sigma_{p}^{2} & 0\\ 0 & 0 & 0 & {I\sigma }_{e}^{2} \end{array}\right]$$

where the matrix A represents the additive relationship between the animals, the matrix I represents the identity, and the variables $\sigma_{h}^{2}$, $\sigma_{u}^{2}$, $\sigma_{p}^{2}$, and $\sigma_{e}^{2}$ represent the variances for herd x year of calving, additive genetics, permanent environment, and residuals. The variance components were estimated using the AIREMLF90 program. The BLUPF90 program was employed to calculate the estimated breeding values (EBVs) and genomic estimated breeding values (GEBVs). 

### 2.3. Estimating the Genetic Parameters

The following formula was used to determine the estimated heritability ($h^{2}$) for the AIREML and ssGAIREML approaches.

$$h^{2}=\frac{\sigma_{u}^{2}}{\sigma_{h}^{2}{+\sigma }_{u}^{2}+\sigma_{p}^{2}+\sigma_{e}^{2}\;}\;$$

Here, $\sigma_{u}^{2}$ defines the additive genetic variance, $\sigma_{h}^{2}$ defines the herd x year of calving variance, $\sigma_{p}^{2}$ represents the permanent environmental variance, and $\sigma_{e}^{2}$ represents residual variance. The accuracies (acc) of the estimated breeding values (EBVs) for both the traditional method and ssGAIREML were determined using the theoretical accuracy approach, which involves the following equation: $acc=\sqrt{1-\left(\frac{{SE}^{2}}{\sigma_{u}^{2}}\right)}$, where ${SE}^{2}$ represents the standard error of variance, and $\sigma_{u}^{2}$ is the additive genetic variance. 

### 2.4. Estimating the Breeding Values

A single-step genomic approach was used to carry out the genomic analysis. The inverse of matrix ***H*** was utilized instead of the inverse of the numerator relationship matrix (***A***) in the mixed-model equations [17], as stated below.

$$H^{-1}=A^{-1}+\left[\begin{array}{cc} 0 & 0\\ 0 & G^{-1}-A_{22}^{-1} \end{array}\right]$$

Here, $A^{-1}$ is the inverse of a pedigree-based relationship matrix for all animals included in the analysis. $G^{-1}$ is the inverse of a genomic relationship matrix (***G***), and $A_{22}^{-1}$ is the inverse of the pedigree-based relationship exclusively for the genotyped animals [32]. The estimated breeding values (*EBVs*) and genomic values (*GEBVs*) were estimated using the BLUPF90 program. The (co)variance components were computed, and their posterior means were used in the analysis, as follows.

$$EBV=\hat{u}$$

$$GEBV={\hat{u}}_{i}+\sum {SNP}_{i}\;$$

### 2.5. Genome-Wide Association Study (GWAS)

The association between individual single-nucleotide polymorphisms (SNPs) and quantitative traits was estimated using the same model as previously employed. The SNP effects were calculated using an iterative procedure identical to that outlined by Wang et al. [33] and implemented in POSTGSF90 software [34]. To identify significant genomic regions associated with the DO, we selected adjacent sets of five SNPs that contributed a minimum of 0.25% to the total genetic variance. These selected regions were subjected to further analyses.

Manhattan plots were generated to illustrate the distribution of important SNPs over the whole genome. The significance level was represented by the negative base 10 logarithm (−log10) of the *p*-value for each SNP [27]. A criterion of −log10 of 5 × 10^−8^ (7.30) was used to identify SNPs that strongly indicated significance. The Manhattan plots for these windows were generated utilizing SAS Studio online v.3.81. To identify potential candidate genes linked to DO, a more detailed investigation was conducted on genes lying within a 37 kb range [35]. Genes were identified utilizing the National Center for Biotechnology Information (NCBI) Map Viewer tool for the bovine genome, with the UMD 3.1 assembly serving as the reference map (https://www.ncbi.nlm.nih.gov/gdv/browser/genome/?id=GCF_000003055.6, accessed on 24 September 2024) [30,36]. For any genes that were discovered, literature and database searches were conducted using the NCBI (https://www.ncbi.nlm.nih.gov/) and GeneCards (https://www.genecards.org/, accessed on 24 September 2024) platforms. Genes located near the windows were utilized to conduct a gene network analysis using the web tool GeneMania [37].

## 3. Results

### 3.1. Comparative Analysis of Days Open by Breed Group and Parity

**Figure 1:**
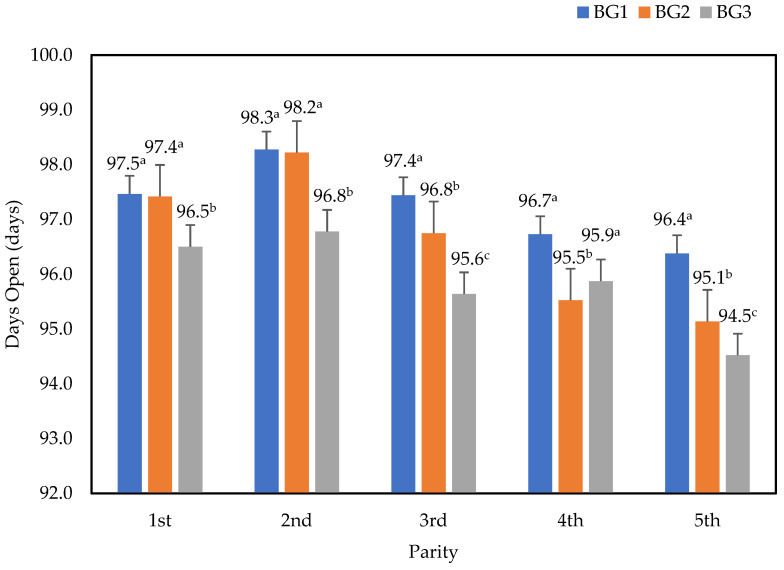
Comparison of the mean values of days open (T, standard error) separated by breed group and parity. The superscripts ^a–c^ indicate statistically significant differences (*p* < 0.05) within each breed group.

Figure 1 compares the days open (DO) for Thai–Holstein crossbreeds, categorized by parity and breed group. The DO values decreased with the decreasing percentage of Holstein genetics in the population. In general, there were significant differences found in each parity for DO (*p* < 0.05). The lowest DO value was found in the fifth parity, and the highest DO value was found in the second parity. The average DO in the BG3 group was the lowest (96.23 days). Conversely, BG1 had the highest average value (97.54 days). There were no significant differences (*p* > 0.05) between BG1 and BG2 groups. 

### 3.2. Analysis of Variance Components and Estimation of Genetic Parameters

Table 2 compares the DO variance components and genetic characteristics between the AIREML and ssGAIREML approaches. The additive variance in the ssGAIREML technique was 20.96 days^2^, which was higher than the additive variance in the AIREML method, which was 18.23 days^2^. In addition, the permanent environmental variance for the ssGAIREML method was lower than that for the AIREML method, and the residual variance in the two methods were nearly identical. The heritability estimates were 0.02 for the AIREML approach and 0.03 for the ssGAIREML method.

### 3.3. Comparison of Accuracy Between Estimated Breeding Values (EBVs) and Genomic Estimated Breeding Values (GEBVs)

Table 3 presents the accuracies of the EBVs and GEBVs for the AIREML and ssGAIREML methods. The datasets were analyzed across multiple scenarios, encompassing the entire dataset, the bull dataset, the dam dataset, and the top 20% of each dataset criterion. The results indicated that the GEBVs obtained through the ssGAIREML method exhibited higher accuracy than the EBVs obtained through the AIREML across all datasets. Notably, the top 20% of the dam dataset for ssGAIREML showed the highest accuracy. The percentage increases in accuracy between the two methods were in the range of 1.64 to 2.76%. The datasets for all animals, bulls, and dams showed greater improvements in accuracy compared to the top 20% subset of each dataset.

### 3.4. The EBVs and GEBVs of Days Open for Thai-Holstein Crossbreeds

Table 4 presents the average EBVs obtained from AIREML and the GEBVs obtained from ssGAIREML for the days open, categorized by dataset and breed group. A negative breeding value for the days open (DO) indicates that the animal has a genetic advantage since it has a shorter interval between calving and becoming pregnant again. The estimates of breed group effects suggest that a lower proportion of Holstein genetics is associated with fewer days open. In addition, the GEBVs obtained from the ssGAIREML approach exhibited more-negative values than those EBVs obtained from the AIREML approach in the top 20% of all the animal, bull, and dam datasets. In these datasets, animals in the BG3 group predominantly exhibited more-negative values, while the BG2 and BG1 groups showed lower negative values. 

### 3.5. Genome-Wide Association Study

**Figure 2:**
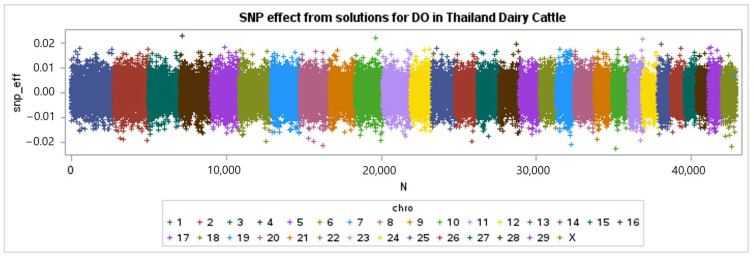
The SNP effect from GEBVs for the days open of Thai–Holstein crossbred cattle.

**Figure 3:**
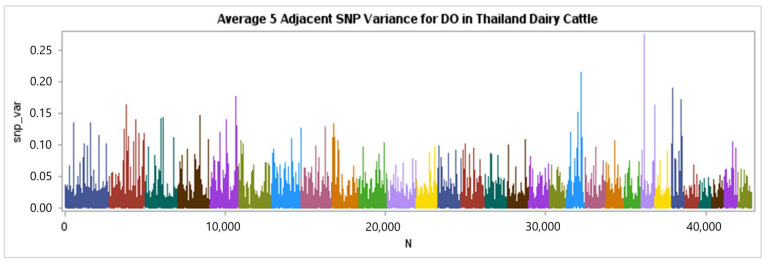
Manhattan plots of the additive genetic variance explained by windows of five adjacent SNPs for DO of Thai–Holstein crossbred cattle. The different colors in the image represent different chromosome names.

**Figure 4:**
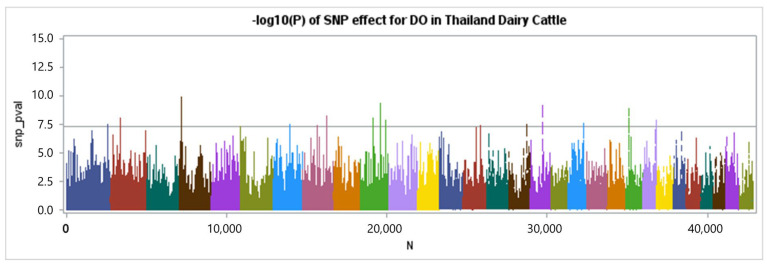
Manhattan plot of GWAS for the days open of Thai–Holstein crossbred cattle. The y-axis depicts the −log10 value of the reported *p*-values for genome-wide SNPs, while the x-axis represents their placements on each chromosome. The horizontal line represents the threshold level, which is suggestive at a significance level of −log10 of 5 × 10^−8^.

The genotypes of 46,852 single-nucleotide polymorphisms (SNPs) were successfully acquired from 868 cows for a genome-wide association (GWAS) analysis. The results were visualized using a Manhattan plot, as shown in Figure 2, Figure 3 and Figure 4. Each color in the plot represents a distinct chromosome number. Following the application of criterion of −log10 of 5 × 10^−8^, (7.30) a total of 16 single-nucleotide polymorphisms (SNPs) were shown to have a significant association with days open (DO) for a total of 13 chromosomes (Table 5).

## 4. Discussion

### 4.1. Comparisons Between Genetic and Genomic Prediction Methods

Days open (DO) is a reproductive trait of significant economic importance commonly used for genetic selection due to the ease with which it can be recorded [5]. This study found that the mean value of the DO for the Thai–Holstein crossbreeds analyzed was 97.02 ± 28.52 days. When comparing data from the same group of dairy cows from 2011, 2015, and 2016, it was found that the DO value had decreased by 56.94% [5,15]. This value was also lower in comparison to other populations, such as Holstein dairy cattle in Iran (117.67 ± 63.60 days) [12], Ethiopian Holstein (107.55 ± 25 days) [10], and Holstein dairy cows in Japan (124.6 ± 67.7 days) [14]. In this study, cows with a Holstein genetic composition of greater than 93.7% (BG1) exhibited the highest DO value among the three breed groups. Holmann et al. [38] stated that a maximum of 85 days is the standard interval for DO to prevent short-term financial losses. An increase in DO from 112 to 166 days leads to daily economic losses of between USD 3.2 and 5.1 per cow on dairy farms in the US [39]. However, dairy farmers at 44 farms in Central Thailand must pay as much as THB 268,088 (approximately USD 8,220.37) for each production period to improve their reproductive performance program [40]. Therefore, farmers might consider raising crossbred dairy cows with more than 87% Holstein genetics as a first step to avoid excessively high DO values. A variety of variables have been examined for their potential impact on the DO, including the Body Condition Score (BCS) at transition or during service, heat stress, age or parity, milk supply, time from calving to first service interval, and peripartum diseases, such as dystocia, metritis, and a retained placenta [41].

The heritability values of the DO assessed using the AIREML and ssGAIREML techniques were 0.02 and 0.03, respectively. The heritability values observed in this study were lower than those reported for the same group in 2015, ranging from 0.03 to 0.04 [42]. Furthermore, the values were lower than those reported for Holstein–Friesian cows in Iran (0.07) [8], Ethiopian Holsteins (0.09) [10], Japanese Holsteins (range from 0.07 to 0.09) [14], Spanish Holsteins (0.04) [9], and Russian Black-and-White cattle (0.07) [43]. The low heritability estimates indicated that the genetic influence on these traits was minimal, and most of the variance in reproductive features among these populations can be attributed to management practices. The low heritability values in this study may result from the significant fixed factors affecting DO and the high residual variance. In previous research conducted by Boonkum et al. [5] and Buaban et al. [15], very low DO values of less than 0.1 were also observed. The cited authors identified factors that influence DO, including differences in Holsteins’ genetic proportions and heat stress induced by hot environments. Researchers in other subtropical countries also found that environmental stress increases the expression of genetic variability [11,44]. Thailand is one of the many countries with a tropical climate that induces heat stress in livestock. In this instance, the modest heritability value for DO is likely primarily due to the variation in the year and month of the calving season among the populations. For this situation, fertility traits can be improved by enhancing reproductive management, preventing heat stress in cattle, employing skilled artificial insemination technicians, utilizing modern milking facilities, and optimizing nutrition [29,45].

In this study, the heritability estimated with the ssGAIREML approach was higher than that calculated with the AIREML method because ssGAIREML estimated greater additive genetic variance. This is also because the ssGAIREML technique uses genomic information, specifically single-nucleotide polymorphism (SNP) markers, to directly measure an individual’s genetic makeup. This approach also enhances the ability to manage incomplete pedigrees using genomic data, thereby mitigating the impact of missing or ambiguous links between animals. By utilizing genomic data, this technique can capture genetic differences not accounted for when solely using pedigree information. Consequently, the enhanced precision acquired leads to a more accurate calculation of genetic variance due to additive effects. In addition, ssGAIREML can use genomic data to consider nonadditive genetic influences, resulting in a more accurate estimation of additive genetic variation. Our findings indicate that the ssGAIREML method was more accurate in identifying GEBVs from genomic selection than the AIREML method (Table 3). Therefore, the ssGAIREML method is an appropriate alternative to the AIREML method in Thai–Holstein dairy breeding programs. It can improve genetic gain, shorten the generation interval, and enable a more accurate selection of high-quality genetic animals. The accuracy of genomic selection in this study was 40.5%. The observed value was lower than those reported in Russian Holstein and Black-and-White cattle (56–93%), and in Canadian Holstein cattle (80%), but higher than in Nordic Red dairy cattle (27 to 31%) [43,46,47]. In another study on Thai Holstein crossbreeds, Boonkum et al. [29] reported that the accuracies of GEBVs for the number of services per conception (NSPC) ranged from 0.49 to 0.52 across all bull and dam datasets. In the Chinese Holstein population, applying GBLUP resulted in a range of 0.59 to 0.76 for the accuracy of GEBVs. According to Ferdosi et al. [48], the accuracy of selecting high-complexity traits, such as reproductive traits, can be influenced by various factors, including the prediction method, the proportion of sires and cow breeds, and the genetic relationship between traits. Hayes et al. [17] found that the accuracy of the genomic BLUP technique decreases rapidly when the connection between the reference and candidate populations diminishes. Hozé et al. [49] has stated that high-density genomics, specifically single-nucleotide polymorphisms (SNPs), have improved the precision of genomic evaluation for small-population breeds that lack reference populations. Increasing the number of genotyped animals in genomic evaluations would substantially improve genetic gain and decrease the rate of inbreeding in the offspring [50]. Hence, a candidate animal that has been genotyped but has no offspring might still provide valuable information for genetic evaluations, which can also impact the estimation of other characteristics [51].

The results in Table 4 indicate that the averaged GEBV was more negative than the GEBV for the top 20% of all animal, bull, and dam datasets. These results suggest that the ssGAIREML method outperformed the AIREML method regarding genetic values. This study’s EBV and GEBV values were lower than those found for Russian Black-and-White cattle populations, averaging between 3.25 and 4.14 [43]. This finding indicates that the Thai–Holstein populations had better genetic values for the DO trait than those populations. The BG estimates showed that a decrease in the proportion of HF genetics results in a decrease in DO. In the three distinct top 20% datasets, animals in BG3 exhibited the most-negative EBV and GEBV values, followed by animals in BG1 and BG2, respectively. This result agrees with the report by Pongpiachan et al. [52], which stated that the reproductive efficiency of purebred cows was much lower than that of crossbred animals despite providing special treatment only to the purebred cows to mitigate the impact of a tropical climate and improving their diet. Nassr et al. [53] also found that crossbred cows exhibited superior reproductive performance regarding Somatic Cell Count (S/C), days open, calving interval, conception at 28 days, mastitis percentage, ketosis percentage, and endometritis. The reduced reproductive efficiency resulting from increased milk production can be substantially restored by implementing more comprehensive health assessments, improved reproductive management, and improved nutrition regimens. It may be necessary to pay more attention to these factors and protect these cows from humid climates to maintain their productivity and reproductive performance in tropical regions.

### 4.2. Identification of Genomic Regions and Candidate Genes

The Manhattan plots of the SNP effect from the GEBVs for the DO are shown in Figure 2. The peaks in the Manhattan plots indicate single-nucleotide polymorphisms (SNPs) that notably impact characteristics. The graphic in Figure 2 displays positive values for the upper portion and negative values for the lower portion. We prefer to select the single-nucleotide polymorphism (SNP) in the lower segment, as a reduced DO value is associated with enhanced reproductive performance [54]. 

The Manhattan plots in Figure 3 display the percentage of additive genetic variance explained by five-SNP moving windows. One window accounted for 0.27% of the total genetic variance in DO, the largest amount of variance explained. Nevertheless, most windows accounted for less than 0.1% of the variation in the traits examined in our study, and these regions with minimal contribution were distributed over the whole genome. This suggests that DO values exhibit a moderate to high level of polygenicity, indicating that multiple areas in the genome contribute to the genetic variance of the traits. The GWAS revealed 16 chromosomal regions pertaining to the DO of Thai Holstein crossbred cattle (Table 5 and Figure 4). The chromosomal areas linked to days-open traits were found on *Bos taurus* autosomes (BTA) 1, 2, 4, 6, 7, 8, 10, 14, 16, 17, 19, 22, and 23. After comparing the results with databases such as NCBI and Genecards, we discovered 28 candidate genes associated with DO. These windows contained 19 identified genes and 9 genes that were not fully described (Table 5). 

The recommended distance between SNPs and genes in the GWAS for cattle is approximately 37 kb to optimize the detection of quantitative trait loci (QTL) while minimizing the effects of linkage disequilibrium (LD) erosion [55]. The findings indicated the presence of eight genes (*DYRK1A*, *CALCR*, *SLC36A1*, *GNA14*, *LOC104973152*, *DPYS*, *RIMBP2*, and *XYLB*) at the target location. Additionally, 14 genes were placed within less than 37 kb, while seven genes were located at a distance greater than 37kb (*LOC107132536*, *S1PR3*, *LOC104973224*, *LOC100140640*, *RIMBP2*, *ACVR2B*, and *SLC22A13*). According to Hajihosseinlo et al. [56], the average r^2^ value decreases with an increase in the distance between SNP pairs, suggesting that SNPs less than 1 Mb apart are more likely to maintain significant associations with QTLs.

### 4.3. Candidate Genes for Days Open

We propose 12 potential candidate genes (*DYRK1A*, *CALCR*, *MIR489*, *MIR653*, *SLC36A1*, *GNA14*, *GNAQ*, *TRNAC-GCA*, *XYLB*, *ACVR2B*, *SLC22A14*, and *EXOC2*) that are believed to have a substantial impact on days open based on position, distance, gene function, and prior references. *DYRK1A* regulates the transcription of genes encoding ribosomal proteins, which are essential for constructing and functioning ribosomes [57,58]. The influence of *DYRK1A* on ribosomal proteins implies its potential effect on reproductive characteristics in cattle, given the crucial role of average cellular growth and function in reproductive processes [59]. Disruption of *DYRK1A* could result in decreased fertility or reproductive efficiency due to compromised cellular processes essential for gamete formation and embryo development. A previous study demonstrated that the excessive expression of DYRK1A in a transgenic mouse model impaired the reproductive ability of male mice by interfering with the initial phases of spermatogenesis [60]. 

*CALCR* encodes the calcitonin receptor, a protein crucial for calcium homeostasis. *CALCR* genes are associated with the functions of neuroactive ligand–receptor interactions, which play a crucial role in maintaining metabolic balance in nursing dairy cows during periods of temperature stress [61]. Previous studies on dairy cattle have shown that CALCR genes are expressed differently in various organs associated with reproductive function and estrus behavior under varying physiological conditions [62]. Hence, this gene can affect the DO of dairy cattle.

*MIR489* and *MIR653* encode microRNAs (miRNAs), which are small non-coding RNA molecules that regulate gene expression. Variations in microRNA sequences can affect interactions with target mRNA in cattle, potentially impacting reproductive capacities [63]. *SLC36A1* encodes the lysosomal amino acid transporter LYAAT1/PAT1 and is expressed in multiple organs. Xia et al. [64] discovered that *SLC36A1* plays a significant role in yak (*Bos grunniens*) mammary tissue and late pregnancy (−15 days).

*GNA14* and *GNAQ* encode proteins as part of the G-protein signaling pathway. These genes involve cellular processes, including signal transduction, cell growth, and differentiation. Chen et al. [65] reported that the *GNA14* gene might play a significant role in animal reproduction, particularly in pigs. The gene’s categorization within the Gq class, known for its participation in signal transduction pathways, suggests possible regulatory functions in reproductive processes. On the other hand, *GNAQ* has been associated with developing ovarian follicles in crossbred beef cattle [66] and endocrine fertility characteristics in Holstein cattle [67]. Furthermore, apart from cattle, this gene has been documented to substantially influence seasonal estrus, reproduction, and litter size in sheep breeds [66,68,69].

*TRNAC-GCA* is a transfer RNA (tRNA) molecule that plays a vital role in protein synthesis. Several previous studies have reported that this gene has an impact on fertility, the production of sperm, the enhancement of sperm quality, and the augmentation of pregnancy rates in dairy and beef cattle [70,71,72]. The *XYLB* and *ACVR2B* genes have been identified as being linked to heifer fertility and activity during estrus, respectively [73,74]. *SLC22A14* has been linked to sperm oxidative phosphorylation and male fertility in mice [75,76]. The *EXOC2* gene is associated with cattle growth and reproduction in pigs [77,78].

## 5. Conclusions

In conclusion, the genetic improvement of reproductive traits remains challenging due to their low heritability values. However, the single-step genomic AIREML (ssGAIREML) method holds promise for enhancing fertility traits. The accuracy of genomic selection is influenced by various factors, including the size of reference populations, the volume of genotypic data, the distribution of genotypes and phenotypes, and how these factors contribute to genomic evaluations. The breed group effect in animals also leads to progressively more negative GEBVs and EBVs for DO as the HF genetic composition decreases. The animals with less than 87.5% HF genetics (BG3) showed the most negative EBV and GEBV values. Twelve prospective candidate genes have been discovered for future usage as selection markers for days open among dairy cattle. This study also suggests that future investigations should use genomic selection and GWAS to assess the effect of heat stress on reproductive traits, particularly in the context of multivariate reproduction.

## Figures and Tables

**Table 1:** The data structure of the days open trait for Thai–Holstein crossbred cattle.

Variable				Data Records
Number of fixed effects				
Herd x year of calving				11,675
Month–year of calving				197
Breed group				3
Parity				5
Number of animals with records (n)				36,368
Number of animals with pedigree (n)				79,071
Number of animals with genotypes (n)				882
Breed groups (BGs)	Average	BG1	BG2	BG3
Number of days open				
Minimum (days)	35	35	35	35
Maximum (days)	150	150	150	150
Mean (days)	97.02	97.54	97.25	96.23 *
SD (days)	28.52	28.86	28.50	28.24
Number of records (n)	59,415	14,776	27,187	17,452

BG1 = less than 87.5% HF genetics, BG2 = 87.5 to 93.6% HF genetics, and BG3 = greater than 93.7% HF genetics; * significantly different (*p* < 0.05).

**Table 2:** The variance components and the heritability (SE) for days open in regard to Thai–Holstein crossbreeds.

Parameters	Methods	
	Traditional AIREML	ssGAIREML
$\sigma_{u}^{2}$	18.23 (3.83)	20.96 (4.28)
$\sigma_{h}^{2}$	30.41 (2.72)	30.35 (2.72)
$\sigma_{p}^{2}$	51.19 (5.56)	48.99 (5.72)
$\sigma_{e}^{2}$	707.87 (6.31)	707.98 (6.31)
$h^{2}$	0.02 (0.001)	0.03 (0.001)

$\sigma_{u}^{2}$ = additive genetic variance, $\sigma_{h}^{2}$ = herd x year of calving variance,$\sigma_{p}^{2}$ = permanent environmental variance, $\sigma_{e}^{2}$ = residual variance, and $h^{2}$ = heritability.

**Table 3:** Comparison between the accuracy of EBVs and GEBVs for the days open for Thai–Holstein crossbreeds using AIREML and ssGAIREML.

**Dataset**	**The AIREML Method**	**The ssGAIREML Method**	**% Increase in Accuracy**
All animals	0.394	0.405	2.76
Bulls	0.394	0.405	2.75
Dams	0.394	0.405	2.75
Top 20% of all animals	0.443	0.455	2.69
Top 20% of the bulls	0.444	0.455	2.68
Top 20% of the dams	0.444	0.456	2.68

**Table 4:** Comparison of breed group effects and breed group averages of EBVs and GEBVs for the days open among Thai–Holstein crossbreeds using AIREML and ssGAIREML.

Dataset	Breed Group	The AIREML Method	The ssGAIREML Method
BG effects	1	0.372	0.327
	2	0.000	0.000
	3	−1.508	−1.412
All animals	1	0.274	0.305
	2	0.273	0.303
	3	0.266	0.299
Top 20% of all animals	1	−1.516	−1.602
	2	−1.501	−1.587
	3	−1.531	−1.620
Top 20% of the bulls	1	−1.516	−1.602
	2	−1.502	−1.588
	3	−1.531	−1.620
Top 20% of the dams	1	−1.515	−1.601
	2	−1.505	−1.591
	3	−1.537	−1.627

Breed Group: 1 = greater than 93.7% HF genetics, 2 = 87.5 to 93.6% HF genetics, and 3 = less than 87.5% HF genetics.

**Table 5:** Significant SNPs associated with the days open of Thai–Holstein crossbred cattle.

No.	SNP	BTA	Location (bp)	−log10 of *p*-Value	Gene	Size (bp)	Distance (bp)
1	ARS-BFGL-NGS-16536	1	151,379,106	7.53	*DYRK1A*	143,395	on target
2	BTB-00088679	2	34,194,876	8.10	*GCA*	20,523	+1808
					*IFIH1*	55,706	−21,954
3	BTB-01831345	4	10,648,384	9.93	*MIR489*	81	−5812
					*CALCR*	110,121	on target
					*MIR653*	89	−5143
4	Hapmap46514-BTA-122322	6	1,091,047	7.32	*LOC107132536*	136,142	−230,032 *
5	Hapmap47414-BTA-79396	7	63,682,321	7.49	*SLC36A1*	85,042	on target
6	ARS-BFGL-NGS-101465	8	53,948,268	7.40	*GNA14*	262,582	on target
					*GNAQ*	307,312	−22,063
7	BTA-52455-no-rs	8	90,808,222	8.27	*S1PR3*	13,229	+145,026 *
8	ARS-BFGL-NGS-17352	10	48,837,583	8.07	*LOC104973152*	206,046	on target
9	Hapmap44851-BTA-76300	10	79,958,340	9.37	*TRNAC-GCA*	71	+33,041
					*LOC104973224*	3091	+71,503 *
10	BTB-01147175	10	95,923,129	7.83	*LOC100140640*	778	−491,941 *
11	BTB-01075462 30517	14	62,361,079	7.44	*DPYS*	90,365	on target
12	ARS-BFGL-NGS-69228 33872	16	71,125,864	7.71	*SYT2*	4935	−22,857
					*LOC104972800*	15,360	−7499
					*LOC104972797*	1244	−24,483
13	ARS-BFGL-NGS-31862	17	47,664,855	9.39	*RIMBP2*	133,098	on target
14	UA-IFASA-6029	19	54,880,876	7.69	*LOC104975129*	12,021	−24,135
15	ARS-BFGL-NGS-24627	22	11,818,533	8.91	*XYLB*	44,159	on target
					*ACVR2B*	30,233	−45,764 *
					*LOC104975492*	12,284	+4783
					*SLC22A14*	13,464	+16,750
					*SLC22A13*	13,362	+41,429 *
16	ARS-BFGL-NGS-30251	23	52,038,120	7.88	*EXOC2*	123,030	+22,058
					*IRF4*	17,252	−9613

BTA, *Bos taurus* autosomes; *, the number that describes the distance between the SNP and the gene is more than 37 kb; +, the location of the SNP is further from the right side of the gene; −, SNP location is further from the left side of the gene.

## Data Availability

Additional data are available upon request from the corresponding authors.
